# Novel Mutation Associated With Papillary Thyroid Cancer

**DOI:** 10.1016/j.aed.2025.04.009

**Published:** 2025-05-08

**Authors:** Deepashree Gupta, Israa Laklouk, Sang Ngo, Masha Livhits

**Affiliations:** 1 Division of Endocrinology, Department of Medicine, David Geffen School of Medicine, University of California, Los Angeles, California; 2 Department of Pathology & Laboratory Medicine, David Geffen School of Medicine, University of California, Los Angeles, California; 3 David Geffen School of Medicine, University of California, Los Angeles, California; 4 Department of Surgery, David Geffen School of Medicine, University of California, Los Angeles, California

**Keywords:** thyroid cancer, molecular markers, mutation

## Abstract

**Background:**

The widespread adoption of molecular testing for cytologically indeterminate thyroid nodules has revealed mutations not previously described in thyroid cancer. The current study reports a novel case of papillary thyroid cancer with a FAT1 mutation.

**Case Report:**

A 47-year-old female presented with a palpable thyroid nodule. Ultrasound revealed a dominant left mid 1.3 cm TI-RADS 4 thyroid nodule. Fine needle aspiration revealed atypia of undetermined significance (Bethesda III); molecular testing with Afirma Genomic Sequencing Classifier was suspicious and identified a FAT1p.V912I c.2734G>A mutation. The patient underwent left thyroid lobectomy. Histopathology revealed papillary thyroid cancer in the index nodule, as well as 2 other foci of papillary thyroid cancer.

**Discussion:**

FAT1 mutation has been previously associated with head and neck squamous cell carcinoma but has not been reported in the context of papillary thyroid cancer.

**Conclusion:**

FAT1 gene variation may be a novel mutation associated with papillary thyroid cancer.

**Highlights**

• PTC is typically driven by BRAF, RAS, RET/PTC point mutations, causing overactivation of the MAPK pathway, accounting for approximately 70% of PTC cases
• PTC with FAT1 mutation did not demonstrate BRAF V600E mutation or high-grade pathologic features, possibly activating MAPK by an alternative mechanism
• This case highlights the importance of considering molecular testing in thyroid nodules, as novel mutations may be detected

**Clinical Relevance**

We are presenting this case of papillary thyroid cancer with a novel mutation. FAT1 mutation has not been described in the literature pertaining to differentiated thyroid cancer. We have described the clinical presentation, fine needle aspiration, molecular marker, and final pathology findings associated with this mutation.

## Introduction

Papillary thyroid cancer (PTC) is the most common form of thyroid cancer, accounting for 70% to 80% of all thyroid cancer cases. PTC is generally indolent with an excellent prognosis. The most common site of spread and recurrence is the cervical lymph nodes. Molecular alterations in the MAP kinase signaling pathway are the most common driver for the development of classic PTC, and mutations in *BRAF V600E* are found in more than 50% of cases.^1^ In contrast, follicular variant of PTC is most commonly associated with a mutation in the *RAS* family. The increasing use of molecular testing for cytologically indeterminate thyroid nodules has provided additional insight into less common mutations that may be associated with thyroid cancer. *FAT1* is a tumor suppressor involved in signaling pathways including MAPK/ERK.^2^ *FAT1* mutations have been previously described in human cancers including squamous cell carcinoma,^3^ but have not been identified in thyroid cancer. This case describes a novel *FAT1* mutation in an indeterminate thyroid nodule confirmed to be PTC on histopathology.

## Case Report

A 47-year-old female with a history of basal cell carcinoma presented to endocrine clinic with a palpable neck mass. Clinically, the patient denied fatigue, weight changes, heat/cold intolerance, bowel/skin changes, or cardiovascular symptoms. She reported no compressive symptoms. There was no history of exposure to ionizing radiation. She was not known to have a family history of thyroid cancer or endocrine disorders because she was adopted. The patient did not have a history of hypothyroidism or hyperthyroidism and was not on thyroid hormone replacement when she presented for her initial thyroid nodule workup. Her thyroid-stimulating hormone was normal at 0.9 mcIU/mL (0.3-4.7 mcIU/mL).

**Fig. 1:**
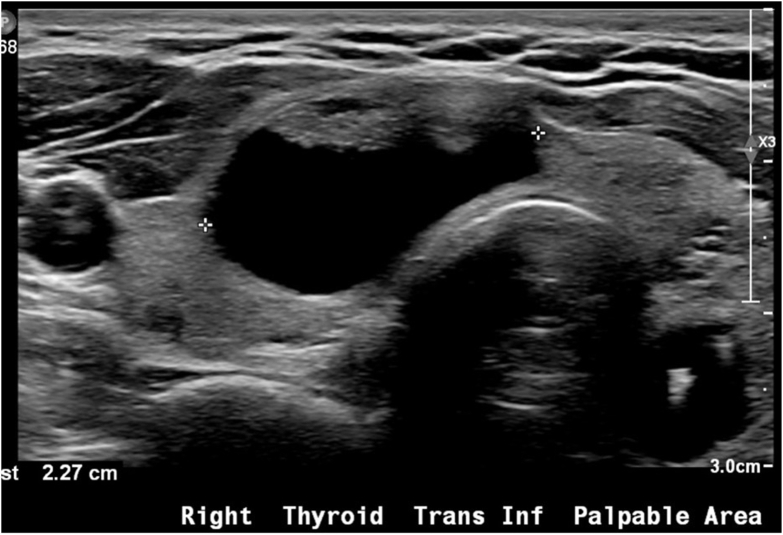
Thyroid ultrasound image of *right* thyroid cystic nodule. The nodule measured 2.3 × 1.3 × 2.3 cm and was TI-RADS 2. It had no internal vascularity.

**Fig. 2:**
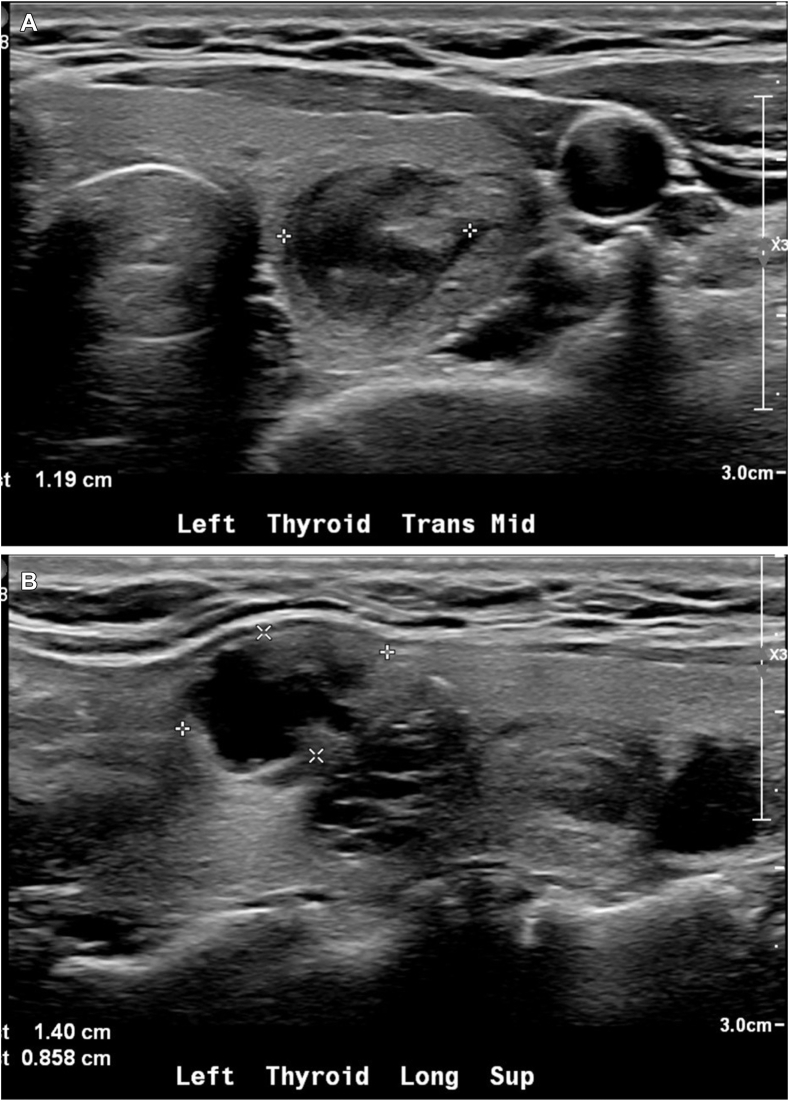
Thyroid ultrasound images of *left* mid thyroid solid and hypoechoic nodule. The nodule measured 1.3 × 1.2 × 1.2 cm and was TI-RADS 4. It did have internal vascular flow. *A*, Antero-posterior view; *B*, Longitudinal view.

**Fig. 3:**
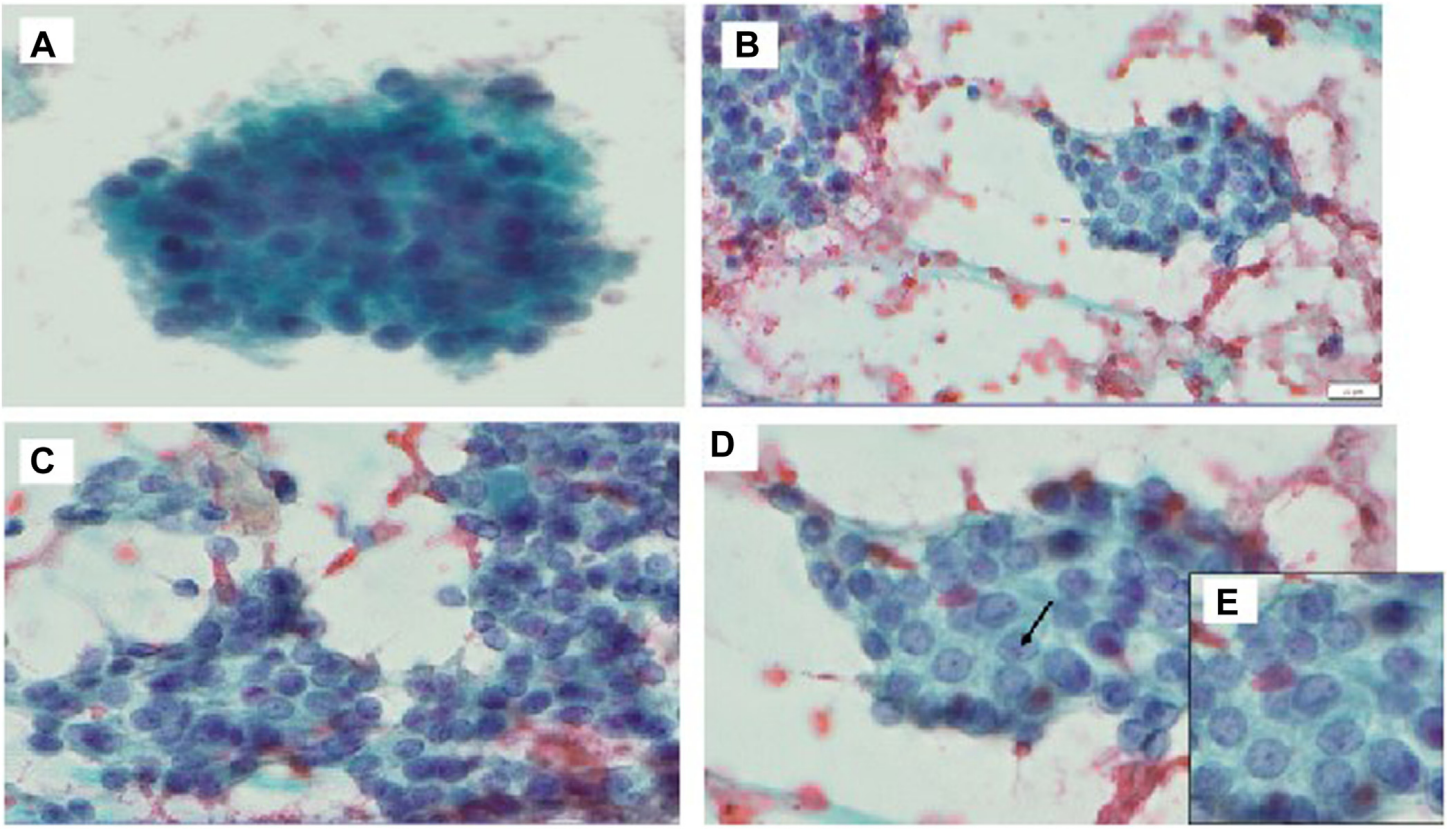
Fine needle aspiration (FNA) images. *A*, FNA shows moderate cellularity composed of clusters of follicular cells forming follicular structure. *B* and *C*, follicular cells exhibiting overlapping, mild irregular nuclear contours, and rare grooving. *D* and *E*, follicular cells exhibiting overlapping, mild irregular nuclear contours, and rare grooving (arrow).

Ultrasound revealed multiple thyroid nodules, including a 2.3 cm mixed cystic and solid right inferior thyroid nodule (Fig. 1), 1.3 cm solid and hypoechoic left mid thyroid nodule (Fig. 2 *A* and *B*), and 1.4 cm mixed cystic and solid left superior thyroid nodule. Fine needle aspiration was performed on the solid left mid thyroid nodule and resulted as atypia of undetermined significance (Bethesda 3) (Fig. 3). Molecular testing with Afirma Gene Sequencing Classifier (GSC) had a suspicious result with a *FAT1*p.V912I c.2734G>A mutation.^4^ Other mutations including *BRAF V600E*, *RET/PTC*, and *RAS* mutations were not identified. The risk of malignancy was estimated at 50% due to the suspicious Afirma GSC result.

**Fig. 4:**
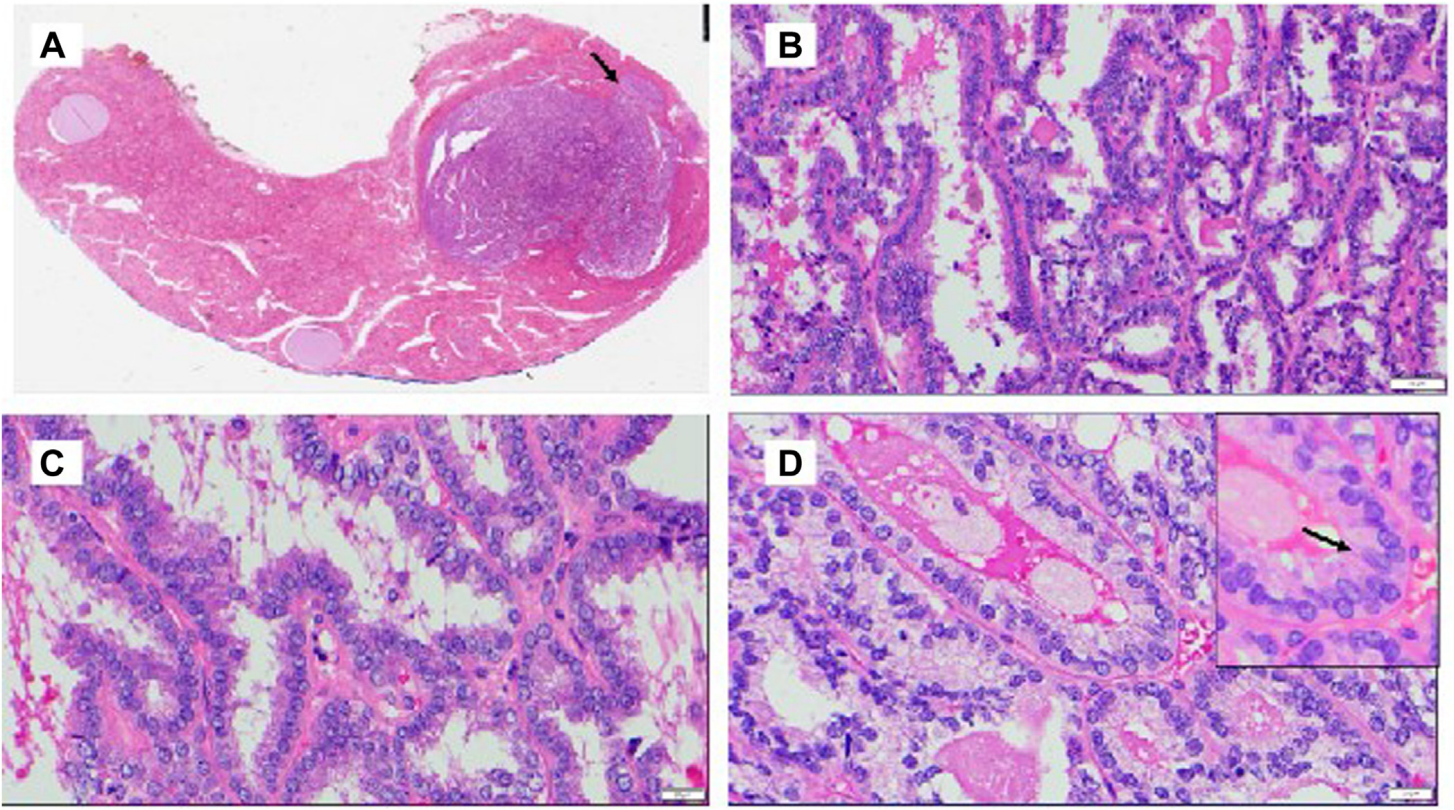
Final pathology images. *A*, Low power view shows a 1.4 cm nodule composed of a well-differentiated follicular cell-derived neoplasm that is well-demarcated but unencapsulated, with a peripheral edge exhibiting significant irregularity (arrow). (1X) *B*, The tumor displays mixed papillary and follicular architectures with a complex anastomosing pattern. (200X) *C*, Short papillary architectures with fibrovascular cores. (400X) *D*, The follicular cells exhibit nuclear features of papillary thyroid carcinoma (PTC) (crowding, elongation of the nuclei, irregular nuclear contours, and occasional grooving (arrow). (400X).

The patient underwent a left thyroid lobectomy. Histopathology revealed 3 foci of PTC (Table). The index solid nodule with the *FAT1* mutation was a 1.4 cm mixed papillary and follicular PTC with no high-grade features such as lymphovascular invasion, tumor necrosis, or increased mitosis (Fig. 4). Immunohistochemistry confirmed a thyroid derived cell lineage with positive PAX8, TTF1, and Thyroglobulin in the index nodule. An additional 1.3 cm follicular variant of PTC was identified in the superior left thyroid lobe, also lacking high-grade features. A third 0.1 cm classic variant of PTC was identified in the superior left thyroid lobe with no high-grade features. Immunohistochemistry was performed for *BRAF V600E*, and only the classic microcarcinoma was positive. The additional tumors could not be sent for *FAT1* testing due to lack of availability of immunohistochemistry. Molecular testing with Afirma GSC can only be performed on indeterminate fine needle aspiration samples, not surgical histopathology.

**Table:** Histopathologic Features of 3 Foci of Papillary Thyroid Carcinoma Identified in Left Thyroid Lobe

Variant of PTC	Location	Size	Molecular alteration	Description
Mixed papillary and follicular	Mid	1.4 cm	FAT1 mutation (by Afirma GSC)	Mixed papillary and follicular architecture, with more than 10% papillary structures present. No high-grade features.
Follicular	Superior	1.3 cm	None	Encapsulated follicular cell-derived neoplasm. No high-grade features.
Classic	Superior	0.1 cm	*BRAF V600E* mutation (identified by IHC)	No high-grade features.

Abbreviations: GSC = Gene Sequencing Classifier; IHC = immunohistochemistry; PTC = papillary thyroid cancer.

## Discussion

We present a case of a patient with multifocal PTC harboring a somatic *FAT1* mutation identified with preoperative molecular testing. The tumor with the *FAT1* mutation did not have aggressive histopathology features. The patient underwent surgical thyroid lobectomy and remains under surveillance.

*FAT1* encodes a protocadherin and is among the most commonly mutated genes in human cancers.^5^ It functions as a tumor suppressor by regulating cell proliferation and migration through signaling pathways including MAPK/ERK, Wnt/β-catenin, and Hippo.^2^ Mutations in *FAT1* can disrupt these pathways and promote tumorigenic processes like epithelial-mesenchymal transition that drive tumor progression.^2^^,^^5^, ^6^, ^7^ Although *FAT1* mutations have been previously associated with squamous cell carcinoma and breast cancer,^3^ their role in PTC has not been characterized.

The pathogenesis of PTC is most commonly driven by point mutations in *BRAF*, *RAS*, or *RET/PTC* chromosomal rearrangements, resulting in overactivation of the MAPK pathway.^8^^,^^9^ These mutations account for approximately 70% of PTC cases.^10^ PTC with a *BRAF V600E* mutation can be associated with more aggressive tumor features, including tall cell variant.^11^ Histopathological analysis in this patient revealed a *BRAF V600E* mutation in the smallest tumor, but it was notably absent from the tumor carrying the *FAT1* mutation. None of the tumors in this case exhibited high-grade features.

Given the involvement of *FAT1* in the MAPK pathway in other cancers, the presence of a *FAT1* mutation in this patient raises the possibility of an alternative route for MAPK activation in PTC. The mechanism by which FAT1 influences MAPK in tumorigenesis remains unclear.^2^ In medullary thyroid cancer, loss of *FAT1* function has been shown to promote cell proliferation, underscoring its role as a tumor suppressor in the thyroid.^12^ In contrast, overexpression of *FAT1* has been shown to suppress oncogenic phenotypes in thyroid cancer cells.^13^

This case highlights the importance of considering molecular testing in thyroid nodules, as novel mutations like *FAT1* may be detected. The detection of rare mutations could have implications for prognostication and the development of targeted drug therapies. Further studies are needed to characterize the significance of *FAT1* mutations in PTC, including their pathophysiology and potential role in tumor aggressiveness, metastatic potential, and impact on patient outcomes.

## Disclosure

The authors have no conflicts of interest to disclose.
